# Oncolytic Viruses—Interaction of Virus and Tumor Cells in the Battle to Eliminate Cancer

**DOI:** 10.3389/fonc.2017.00195

**Published:** 2017-09-08

**Authors:** Anwen Howells, Giulia Marelli, Nicholas R. Lemoine, Yaohe Wang

**Affiliations:** ^1^Centre for Molecular Oncology, Barts Cancer Institute, Queen Mary University of London, London, United Kingdom; ^2^National Centre for International Research in Cell and Gene Therapy, Sino-British Research Centre for Molecular Oncology, Academy of Medical Sciences, Zhengzhou University, Zhengzhou, China

**Keywords:** oncolytic virus, tumor cells, selectivity, cancer treatment, host factors

## Abstract

Oncolytic viruses (OVs) are an emerging treatment option for many cancer types and have recently been the focus of extensive research aiming to develop their therapeutic potential. The ultimate aim is to design a virus which can effectively replicate within the host, specifically target and lyse tumor cells and induce robust, long lasting tumor-specific immunity. There are a number of viruses which are either naturally tumor-selective or can be modified to specifically target and eliminate tumor cells. This means they are able to infect only tumor cells and healthy tissue remains unharmed. This specificity is imperative in order to reduce the side effects of oncolytic virotherapy. These viruses can also be modified by various methods including insertion and deletion of specific genes with the aim of improving their efficacy and safety profiles. In this review, we have provided an overview of the various virus species currently being investigated for their oncolytic potential and the positive and negative effects of a multitude of modifications used to increase their infectivity, anti-tumor immunity, and treatment safety, in particular focusing on the interaction of tumor cells and OVs.

## Introduction

One of the most promising developments in cancer therapy to emerge over the past few decades is oncolytic virotherapy (OVT). Many of the more traditional treatment options routinely used to combat cancer in the clinic are not efficacious enough and have considerable side effects for patients. Although these treatments, such as chemotherapy and radiotherapy, are advancing and becoming more tolerable, we are yet to discover an alternative treatment option that has a high level of potency with minimal side effects that will dramatically change the overall survival of cancer patients.

**Figure 1:**
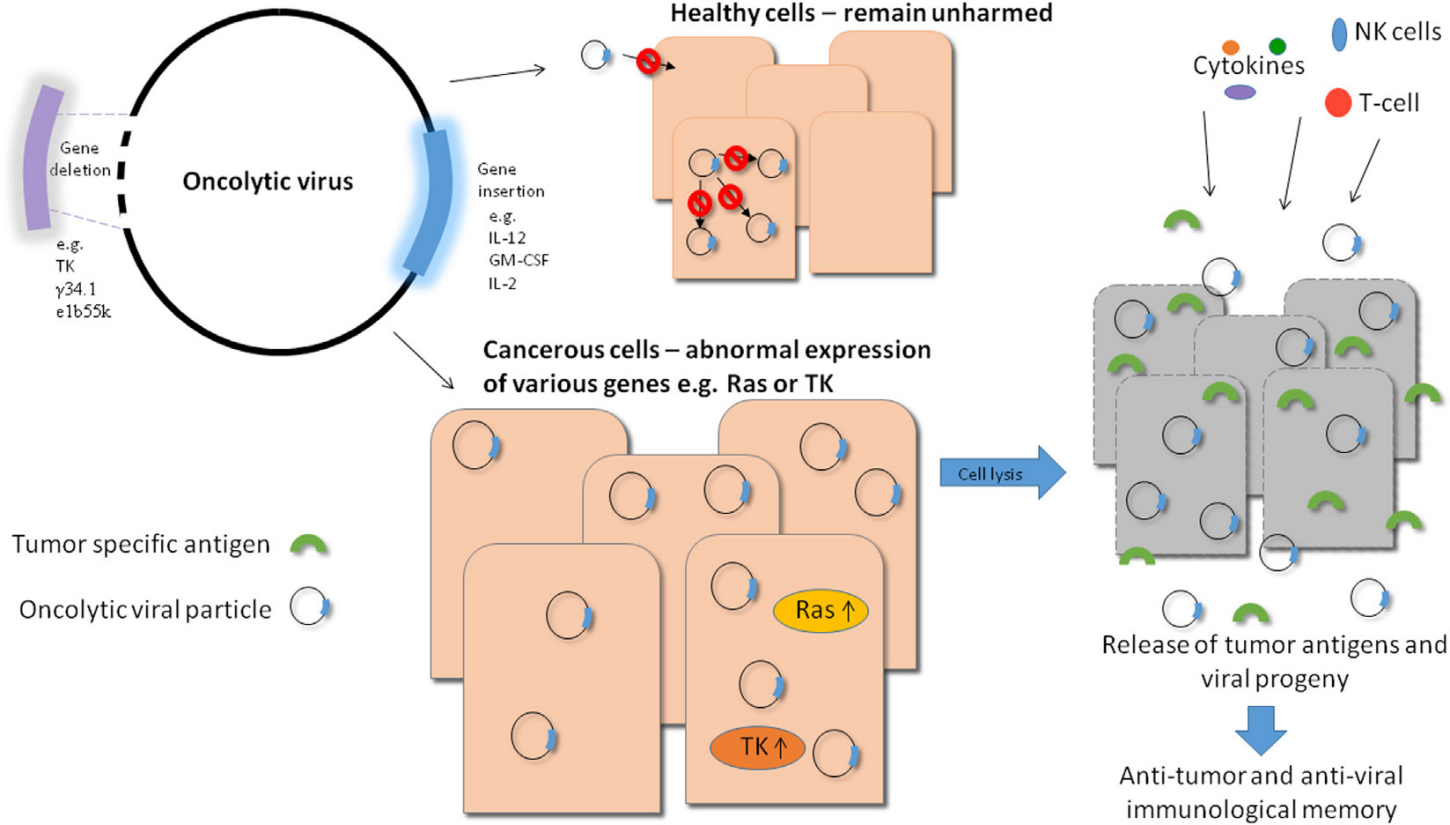
Overview of oncolytic viral therapy. Genetic engineering has made it possible to modify oncolytic viruses (OVs) to make them safer and more effective against tumor cells. Various genes can be deleted to produce a safer virus, e.g., thymidine kinase (TK), while others can be inserted into the viral genome to increase efficacy, e.g., immune stimulators like IL-12. By carrying out these modifications, OVs can be made safer as they require target cells to provide the essential deleted gene, e.g., TK which is upregulated in tumor cells and not in healthy cells. They can also generate long lasting immunity as a result of stimulation of a potent immune response in which tumor cell antigens (along with viral antigens) can be targeted by T-cells. Also, when the virally infected tumor cells die, they release progeny virions into the tumor microenvironment which can in turn infect neighboring cells, improving the efficiency of viral treatment. Systemically delivered OVs also have the potential to eliminate metastatic tumor cells.

Oncolytic viruses (OVs) have the potential to deliver this goal and much effort has been put into improvement of their efficacy and safety profiles in recent years. There are numerous viruses which either have naturally oncolytic properties or have been engineered to specifically lyse tumor cells. The great advantage of this therapy is that these viruses are able to specifically target tumor cells and therefore healthy tissue is not damaged during the course of the treatment. There are various ways to improve the specificity of OVs, for example, taking advantage of pathways which are upregulated in tumor cells and not healthy cells and engineering a virus which relies on such a pathway for successful infection thereby rendering the virus incapable of infecting healthy tissue (see Figure 1).

Another factor of oncolytic viral therapy that makes it a promising candidate is that while viral infection can directly lyse tumor cells, the resultant immune response will be generated not only to viral antigens but also to tumor cell antigens. This unique feature of oncolytic viral therapy makes it a very exciting avenue of research as not only can the viruses act to eradicate existing tumors but they can also potentially generate lasting immunity in the form of memory T-cells which are primed against tumor cell antigens (see Figure 1).

In addition to causing direct lysis of cells, OVs can also be used as delivery vectors for therapeutic genes. In this case, the virus can be genetically modified to include the gene of interest which upon infection and viral replication will be produced at high levels in infected tumor cells where it can exert its function. There are a multitude of anti-cancer genes that can be incorporated into OVs in this way in order to maximize the efficacy of the virus and improve the anti-tumor response generated (see Figure 1).

Also, OVs have great potential as combination therapies used together with more traditional approaches. In this way, various treatment options can be used synergistically to combat cancer from more than one angle at a time which will likely give rise to a more positive response to treatment. This combination therapy approach can lead to improved tolerance of treatment in patients as the synergistic effect allows lower doses of each individual therapy to be used to gain similar effects compared with the use of one treatment alone.

As stated, much research effort has been put into improving this area of cancer therapy and the various viruses used and important advances made in their development are discussed here.

## History of OV Therapy

The use of OVs was first conceived following the observation of the fact that during or after an infection, tumor regression is occasionally observed (1–3). Based on this observation, patients with Hodgkin’s lymphoma were treated with serum containing hepatitis virus (4).

In the following years, a lot of effort was put in to achieving better and safer results. For 30 years, from 1950 to 1980, many studies were performed without reaching good clinical outcomes or providing long-term results (5–7). This was mainly due to the fact that viral treatments were unsafe because there were no methods to control virulence nor to obtain tumor specificity. Finally, in the late 1980s, with the advent of genetic engineering, a renewed interest for OVT rose again and in recent years many advances have been made in this field.

## Selectivity of OVs

There are various ways in which different OVs are able to infect cells. Some viruses, like vaccinia virus (VV) or Newcastle disease virus (NDV) lack specific receptors for attachment so enter cells *via* endocytosis. Other viruses have a specific receptor that they use to enter host cells; for example, adenoviruses (Ads) are able to bind coxsackie and adenovirus receptor (CAR), integrins, or cluster of differentiation 46 (CD46). Measles can also use CD46 for entry, whereas herpes simplex virus (HSV) uses nectin or herpesvirus entry mediator (8, 9). Despite the observed tendency of tumor cells to upregulate some of these receptors, they are also expressed on many normal cells.

There are a variety of ways in which OVs can be targeted to tumor cells in order to minimize damage to healthy cells. These include exploitation of various pathways which are aberrantly expressed in tumor cells to ensure engineered viruses are only capable of productive infection in cells which have abnormal levels of certain genes. Also, control of viral replication with microRNA differentially expressed in tumor cells compared with healthy cells can restrict viral replication specifically to tumor cells. Viral coat proteins can also be manipulated to ensure viral infection only occurs in cells with certain receptors, e.g., receptors found on tumor cells only. These strategies will be discussed in more detail here.

As it is essential that OVs only successfully infect tumor cells to avoid the spread of virus in healthy tissue, many different approaches have been investigated to increase specificity. One of these is to take advantage of the aberrant expression of various proteins in pathways which can have an effect on viral replication. Of these pathways, OVs commonly exploit aberrant expression of proteins involved in the Ras pathway. This pathway is generally silent in normal cells but activated in tumor cells and the downstream effects of this can be beneficial for OV infection (10, 11). There are a number of ways in which upregulation of the Ras pathway in tumor cells can influence the outcome of oncolytic viral infection. For example, it has been shown that the Ras/MEK pathway can downregulate specific interferon-inducible genes which may have an effect on anti-viral responses and apoptosis control (12). It has also been seen that apoptosis can be involved in the increased efficiency of OVs in tumor cells. In the case of Reovirus, this OV can cause an accumulation of Ras within the Golgi body which leads to triggering of apoptosis signaling pathways and subsequent release and spread of progeny virions (13).

Because of aberrant expression, genes involved in the Ras pathway (among others) can favor replication of viruses in tumor cells and many viruses have been engineered to exploit this to increase their selectivity for transformed cells. For example, engineering viruses which are only able to express certain critical viral proteins upon upregulation of transcription factors downstream of the Ras pathway renders the virus only able to replicate in cells with an upregulated Ras pathway (14).

Other strategies used to produce tumor-targeted replicating OVs include control of certain genes using microRNA. Hikicki et al. have shown that it is possible to place critical viral genes under the control of an miRNA which has low expression levels in tumor cells. This renders the virus unable to successfully infect healthy cells where normal levels of this miRNA are expressed, facilitating interference with production of the critical viral gene (15).

Also, modification of viral coat proteins can be used to specifically direct viral infection to tumor cells. There are various ways to achieve this, for example, covering the viral surface with polymer to “cloak” the existing receptor and addition of epidermal growth factor (EGF) to target the virus to tumor cells which tend to have upregulated EGF receptor (EGFR) expression (16). This strategy not only reduces the broad tropism conferred by the existing viral receptor but also replaces this with tumor specific receptors to direct oncolytic viral infection to target cells, leaving healthy tissue unharmed.

Another approach involves the use of antibodies to target OVs to tumor cells. As an example of this strategy, it was found by Watkins et al. that antibodies can be engineered which contain an Ad fiber protein targeting single-chain variable fragment (scFv), linked to EGF. This facilitated targeting of Ad to EGFR-upregulated tumor cells (17). This antibody focused approach was further developed to allow incorporation of scFv into the viral envelope. For example, HSV type-1 (HSV-1) relies on various glycoproteins for entry into cells and one of these glycoproteins (gD) is responsible for interaction with the viral entry receptors. If an scFv targeting EGFR is fused to this glycoprotein, the virus is then able to use EGFR as an entry receptor which improves tumor targeting (18).

In parallel to the attempt to create safer viruses, a new strategy is developing with the aim of incorporating transgenes within the virus to target the tumor microenvironment or to activate the immune system.

## Modifications of OVs

This new strategy is achieved by modification of viral genomes by insertion or deletion of selected genes which aid or hinder oncolytic potential and some of the strategies being explored will be discussed in more detail here.

The ability to modify the genome to our advantage is one of the most promising aspects of OVs. These modifications have a variety of functions including improvement of tumor tropism and increased recruitment of the immune system to aid anti-tumor responses. Also, improved safety and efficacy of OVs are of utmost importance and are another aspect that can be controlled by genetic engineering. Some examples of gene editing include deleting replication-related genes to reduce replication efficiency (as attenuation improves safety) and/or addition of genes that induce pathways to promote tumor cell death, for example, the apoptosis pathway (19).

As previously mentioned, pathways which are alternatively regulated in tumor cells compared with healthy cells can be exploited to produce selective viruses. For example, genes can be deleted resulting in a virus that can only successfully infect certain tumor types which over-express MEK (20). This strategy can also be used with HSV whereby genes can be deleted to produce a virus which preferentially replicates in tumor cells which unlike healthy cells tend to have a constitutively activated Ras pathway (21). As this virus initiates apoptosis in infected and bystander cells and preferentially infects tumor cells, it can be used as oncolytic agent with this deletion (21, 22).

## Gene Deletion Strategies

Many OVs have been modified with specific gene deletions to target the virus to tumor cells and inhibit infectivity in healthy cells. An example of this strategy is thymidine kinase (TK) deletion from VV. As the wild-type virus usually encodes this kinase it is able to replicate in healthy cells, however, when the gene is deleted the virus can no longer replicate efficiently in healthy cells. As tumor cells produce higher levels of TK, even though the gene is deleted the virus is still able to replicate in these cells (23, 24).

Another strategy used to improve tumor specificity is to delete apoptosis-inhibiting genes (usually used with Ad). In wild-type infections, Ad encodes genes which block apoptosis which is an advantage as infected cells then become viral factories given that they will not enter apoptosis in response to infection. As tumor cells often block apoptotic pathways as a survival mechanism, Ads with deleted apoptosis-inhibiting genes can undergo prolonged infection while infection of healthy cells will lead to lysis of the cell and clearance of virus (25).

Gene deletion strategies can also be used to improve the efficiency of virus delivery systems which are designed to deliver OVs to target cells without interference from the host immune system. In these systems, viruses are delivered within host cells (e.g., mesenchymal stem cells) that provide shelter from immune attack and subvert the problem of clearance of virus (by neutralizing antibodies) before they reach their target cells. This method has been improved in Ad by modification of the virus in order to make it more infective in MSC and more efficient at killing tumor cells. Oncolytic Ad can also be engineered through deletion of an anti-apoptotic gene, improving virus release from MSC, and allowing more potent anti-tumoral activity (26).

Although gene deletion often improves efficacy of OVs, the chosen candidates need to be selected very carefully. Various gene deletions have the potential to alter viral infectivity in ways which can either improve or diminish oncolytic potential. For example, deletion of E3-6.7K/gp19K leads to more rapid viral clearance which on one hand improves safety but on the other hand only allows a short time-frame for inserted therapeutic genes to be delivered to target cells and produced in a significant amount. Therefore, this particular deletion can only successfully be used for delivery of genes which can act quickly to have the desired effect (27). Another study has shown that deletion of a combination of viral genes will enhance tumor selectivity but reduce viral potency, highlighting the problems faced in engineering the perfect oncolytic viral therapy (28). There are also genes encoded by OVs that can act to inhibit oncolytic potential, for example, E4orf1 encoded by Ad leads to increased levels of survival in infected cells thereby reducing the ability of the virus to directly lyse infected tumor cells (29). This effect can lead to diminished efficacy and therefore needs to be addressed in order to maximize the potential of this virus to treat cancer.

## Gene Insertion Strategies

Another advantage of gene deletion is the opportunity to insert therapeutic genes in their place without disrupting the reading frame (30). There are a multitude of therapeutic genes and immune stimulators which can be delivered within OVs to combat cancer (for example, the interleukin family of genes used to stimulate the immune system thereby improving anti-tumor immune responses). However, this approach is not perfect and the combination of deleting a gene and inserting a new one can result in problems of its own. For example, deletion of the E1B55K gene leads to improved virus spread (as it facilitates apoptosis), however, this may result in low levels of production of the inserted gene as the cell undergoes apoptosis before high quantities of the gene are expressed. The combinations of deletion and insertion need to be specifically studied in order to ascertain which ones complement each other and which have negative effects on each other (31, 32). Addition of cytokines or other genes may also give rise to toxicity, for example, the IL-12 cytokine has been seen to result in side effects that cause a poor safety profile. This problem has been combated in a range of ways with varying results; these include using a single-chain version of the cytokine and anchoring IL-12 to the membrane of cells through fusion with the CD4 trans-membrane region. These methods did not produce the desired reduction in toxicity without reducing anti-tumor efficacy, however, using a helper-dependent Ad vector with an inducible expression system was successful in allowing production of IL-12 without high levels of toxicity (33). Another strategy to reduce potency of the IL-12 cytokine is to deliver it within conditionally replicative Ad rather than replication competent strains. For example, on delivery within an adenoviral vector which can only replicate in hypoxic conditions typical of tumor masses, IL-12 was still effective but resulted in less toxicity and more specific delivery to target cells compared with replication competent viral delivery (34).

## Other Strategies to Improve OVs

As well as addition and deletion, control of gene promoters can be used to modify viral behavior. For example, promoters that are activated more highly in tumor cells can be used to control an essential viral gene rendering that virus incapable of replicating in healthy cells (32). They can, however, replicate successfully in tumor cells as these cells have a higher activation of that promoter (e.g., use of Cyclin E promoter to target Ad infection to tumor cells) (35). It is also possible to use promoter control of virus genes in order to attenuate virus and make the therapy safer. If genes essential to virus replication are expressed under the control of a promoter downregulated in tumor cells, then delivery of such a tumor-selective virus into those cells will only allow a low level of infection which results in improved safety with retention of oncolytic activity (although at a lower level than wild-type infection) (15).

A constantly evolving area of research is the combination of OVs with other treatments for synergistic effect. For example, combining oncolytic Ad with a cytotoxic drug currently used in the clinic enhanced the anti-tumor efficacy of this treatment (36). These combinations can improve the activity of OVs in various ways, including promoting better replication or compensating for certain deletions without compromising tumor selectivity. For example, if a deleted gene has more than one function, it can be eliminated to improve selectivity in combination with delivery of a compound that can be administered to improve replication efficiency which may have been lost through deletion; an example is the use of 2-aminopurine to enhance the oncolytic activity of an E1B-deleted Ad (37). Combining treatments can also facilitate administration of each agent at lower and safer doses given their synergistic effect (38). Also, the combination of oncolytic Ad with CAR upregulation can improve the efficacy of this treatment due to the use of these receptor molecules for Ad infection (39). Various tumor cell types express different levels of CAR and this will have an impact on the ability of the virus to effectively treat different tumors. Therefore, administration of an agent which can upregulate CAR expression before oncolytic Ad therapy could increase the efficacy of adenoviral treatment.

There are numerous viruses which have been found to have natural or engineered oncolytic activity and some of the most promising candidates will be explored in more detail in the following sections.

## Vaccinia Virus

Vaccinia virus is a naturally oncolytic virus which was found to have a natural tropism for tumor cells due to its sensitivity to type I interferon (40). It is a double-stranded DNA virus of the *Poxviridae* family. There are many different strains of the virus and of these; Lister, Wyeth, and Western Reserve strains are the most used in research. VV is a very promising anti-cancer agent (41) for many reasons including its very short life cycle (around 8 h) and its ability to replicate in hypoxic conditions (42). Moreover, it does not have a specific receptor and viral fusion with the plasma membrane facilitates entry (43) which makes it a potential candidate for treatment of all tumor types. Furthermore, VV does not depend on the host cell for mRNA transcription and its entire life cycle takes place in the cytoplasm, eliminating the risk of genomic integration (44). The virus, which can infect both human and mouse cells, is infectious at four different stages of its life cycle: intracellular mature virion released by cell disruption, intracellular enveloped virion, cell-associated enveloped virion, or extracellular enveloped virion released by endocytosis from the membrane.

To make oncolytic VV safer, two deletions have been made; one in the TK region (45) (as for HSV) and one in the vaccinia growth factor gene region increasing its specificity for tumor cells (23). As well as this, many efforts have been made to generate a strain that results in viral attenuation, rendering the virus harmless in normal tissue. For this purpose, mutations in the F14.5L and A56R genes have been engineered. The F14.5L gene encodes a secretory signal peptide, while the A56R gene generates hemagglutinin.

In addition to these safety measures, many different genes have been integrated into the VV genome in order to increase its anti-tumor efficacy such as cytokines and antibodies, as reviewed by Badrinath et al. (9). As an example, JX-594 is a VV with TK gene deletion and GM-CSF (a cytokine able to stimulate the immune system to kill tumor cells) gene insertion and is currently undergoing phase III trials. Phase I trials have shown promising results and acceptable safety profiles (46) and phase II studies were designed to investigate the optimal dose of intravenously delivered JX-594 (47). The results of a randomized, dose-finding phase II clinical trial reported by Heo et al. showed that high dose JX-594 resulted in higher overall survival duration than low dose administration in patients with advanced hepatocellular carcinoma. This study also demonstrated both anti-viral and anti-tumor immunity generated in clinical patients in response to OV administration (48).

There are many other modifications which can improve the anti-tumor efficacy of VV. For example, the addition of IL-10 was found to improve the oncolytic activity of VV through dampening of anti-viral immunity (prolonging viral infection) without reducing anti-tumor immunity (49).

The major concern in using VV as oncolytic therapy is the fact that it is easily recognized by the immune system. Indeed, the strains of VV exploited in clinical OVT are derived from the vaccine formulations used for smallpox eradication and so there is an activation of the immune system in those patients that were administered with the vaccination. Despite this, Breitbach et al. demonstrate in a clinical study that the intravenous administration of the modified VV JX-594 was safe and that the virus was able to replicate and to express the transgene only in tumor cells (50).

## Adenovirus

Adenovirus is one of the most commonly studied viruses in oncolytic therapy and was the first to be given regulatory approval, granted by the State Food and Drug Administration in China in 2005 (51). It is a non-enveloped, double-stranded DNA virus of the *Adenoviridae* family. There are several strains of Ad and of these, Ad5 is the most commonly used in oncolytic therapy. As this virus is widely studied, there are a multitude of various modifications which have been shown to improve its efficacy and safety which will be further discussed below.

It has been seen that addition of various genes to oncolytic Ads can improve their anti-tumor efficacy. For example, the combination of p53 addition to suppress tumor growth with GM-CSF addition to induce the apoptotic pathway elicits a synergistic effect which is effective in combating hepatocellular cancer stem cells (52). This is especially exciting as it potentially provides a mechanism to combat cancer stem cells which are considered to be integral to cancer recurrence after current treatment options. GM-CSF addition has been tested in phase I clinical trials and was shown to be non-toxic and tolerable at the doses used, however, the efficacy showed room for improvement in terms of anti-tumor efficacy and long-term immunity (53).

It is also possible to improve oncolytic Ad by incorporation of a short-hairpin RNA which functions to downregulate Dicer (an endoribonuclease which has a role in processing virus-associated RNA). Downregulation of this protein inhibits the destruction of viral RNA and allows Ad to replicate efficiently and therefore improves the efficacy of this OV (54).

Gene silencing techniques can also be used in order to downregulate certain oncogenes in order to suppress tumor growth. For example, downregulation of EphA3 by insertion of siRNA targeting this gene into the genome of an Ad whose replication is made conditional under the control of TERTp (which increases specificity for tumor cells) results in increased levels of autophagy through inhibition of the AKT/mTOR pathway. This allows the virus to both inhibit tumor cell proliferation and kill infected cells (55).

In terms of important features of OVs on which to focus research efforts, it has been shown that the T-cell immune response to oncolytic adenoviral infection is more efficacious in combating tumors than direct lysis of tumor cells by viral infection (56).

It has been previously shown that treatment of tumors with immune checkpoint inhibitors (e.g., PD-L1 blockade) allows immune-boosting viral treatments to have a longer lasting effect. It has recently been found that encoding a PD-L1 blocking antibody within Ad vectors leads to production of this antibody in the local vicinity of tumors by host cells and this approach gives rise to better anti-tumor effects than with infusion of the antibody into tumors. When combined with chimeric antigen receptor-modified T-cell treatment targeting HER2 positive tumor cells, this approach to PD-L1 antibody delivery increased treatment efficacy (57).

In addition to investigating methods of improving current Ad strains used in oncolytic therapy, it is also important to find potentially new strains which may prove to be more appropriate for therapy. For example, Ad11 was found to have higher levels of receptor availability and lower levels of neutralizing antibody than Ad5 (which is the most commonly utilized strain in OV engineering). When this strain is modified to replace its E1A enhancer-promoter region with that of Ad5 (leading to higher levels of E1A mRNA) it can become a more potent OV. With additional modifications to increase tumor specificity, the Ad11/Ad5 strain could prove to be a more successful OV than the more commonly studied Ad5 strain (58). Also, another group B oncolytic Ad is enadenotucirev, this virus is able to infect both at apical and basolateral surfaces of polarized cells and results in progeny being released *via* the apical surface which directs them into the tumor mass rather than out into the blood stream (59). This proves to be an improvement on the traditional Ad5 strains used in the design of oncolytic therapy as type 5 Ads seem to infect preferentially *via* the apical surface which could pose a problem when delivered systemically.

Even once a promising candidate has been identified for oncolytic therapy, one of the major obstacles to effective use of OVs is clearance by the host immune response (namely anti-viral T-cells) but it would be extremely useful to use this response to our advantage. In a recent study, it has been found that it is possible to engage and therefore redirect these T-cells to react to specific antigens (in this case EGFR which is often overexpressed on tumor cells) in order to redirect the anti-viral response into an anti-tumor response (60). Also, the generation of T-cell responses both to virus and tumor was found to be more important in viral efficacy than direct oncolysis (56). Therefore, a balance between increasing T-cell production to improve anti-tumor immunity and controlling T-cell response to reduce viral clearance is necessary.

In the constant effort to find new and improved oncolytic therapies, it was found that certain cancer cell types produce a peptide which inhibits the ability of the Ad to escape endosomes and be released (61). This inhibition presents a barrier to effective viral spread and successful oncolytic therapy. However, type 3 Ad has evolved to manufacture a decoy capsid which sequesters HD5 and renders the virus able to escape endosomes (62). This finding suggests that the mechanism used by this strain of Ad can be mimicked in order to improve efficacy of existing or potential oncolytic Ad therapies.

Another obstacle facing oncolytic therapy is the tumor microenvironment and its immuno-suppressive properties. For example, expression of TGF-β in the tumor microenvironment results in diminished ability of virally delivered IL-12 to boost anti-tumor immune responses. One method to overcome this problem is to co-express decorin which leads to attenuated TGF-β expression in tumors (63). This is just one example of therapeutic gene addition which improves the action of oncolytic Ads armed with cytokines aimed at boosting anti-tumor immune responses.

Combination therapy, whereby OVs are used in combination with conventional therapy is a growing area of research with many promising leads. For example, it has been shown recently that Ad encoding pro-inflammatory IL-18 cytokine has a synergistic effect when delivered in combination with dacarbazine, which is conventionally used to treat melanoma by alkylation of DNA strands. When used in combination, it was seen that these treatments together result in inhibition of tumor cell growth and increase in apoptosis (64). A similar strategy was employed in another study whereby IL-12 was used as an immune stimulator and a VEGF-silencing ribonucleic acid was co-expressed in order to overcome the immune suppressive action of VEGF produced by tumor cells (65). Many studies have been undertaken to assess which combinations work best and promising results have been reported, for example, oncolytic Ad combined with Temozolomide treatment (alkylating agent) leads to increased levels of viral replication and tumor cell death *via* various mechanisms thought to include upregulation of autophagy and apoptosis pathways (66). Another combinatorial approach to cancer treatment is to combine OV with anti-tumor antibody treatment. It was found that the gene for an anti-HER2 antibody used in the clinic (Trastuzumab) to combat HER2 positive breast cancers could be inserted into an oncolytic vector and successfully translated into antibody within tumor cells. This was then released upon lysis of infected cells (aiding treatment specificity) and provided an answer to some of the difficulties in delivery of antibodies systemically, specifically the infiltration of tumor masses as virus can infect, and spread throughout the tumor delivering the antibody with it (67).

Another approach to combination therapy is to use two antigenically distinct OVs sequentially (whereby the second to be delivered is not cleared by immunological memory to the first dose). This strategy was reported to be successful when oncolytic VV was used after first administering adenoviral therapy. In this case, it was seen that the increased efficacy was dependent on T-cell activity (68). Subsequent to this finding, various other regimes have also been found to improve the efficacy of OVs. For example, sequential delivery of oncolytic Ad and NDV which are both engineered to express an immuno-stimulatory cytokine leads to significant anti-tumor responses even though when administered alone, each virus showed limited efficacy against tumors (69).

Another exciting avenue being explored to improve the oncolytic potential of Ads is to combine oncolytic therapy with induction of the autophagy pathway. It was found that this pathway is involved in viral antigen presentation and therefore its upregulation could increase presentation of virally delivered tumor-associated antigens (TAAs) at the cell surface in order to induce a more potent anti-tumor immune response than with antigen delivery alone (70).

Existing neutralizing antibodies pose yet another problem in the design of OVs. Certain viral strains may have been previously encountered/vaccinated against and therefore neutralizing antibodies can be rapidly produced by the host upon treatment (especially when virus is delivered intravenously) and clear the virus before it can have an effect on tumor cells. To combat this problem, it was found that coating virus in albumin provided a protective barrier from neutralizing antibodies in the host and therefore facilitated systemic delivery without the threat of viral clearance due to previously generated immunological memory (71).

One of the main barriers to viral spread in tumor masses is the interstitial matrix (including extracellular DNA). OVs have therefore been modified to encode proteins which will degrade the interstitial matrix along with expression of DNaseI to degrade the extracellular DNA, therefore allowing more efficient spread of OV throughout tumor masses (72).

Oncolytic viruses can also be exploited as carriers for anti-cancer drugs using more than one method. In a recent study, it was found that electrostatic attraction between viral capsid and the drug molecules themselves was an efficient way to deliver anti-cancer drugs which would then act synergistically with the oncolytic adenoviral therapy (73).

## Herpes Simplex Virus

Herpes simplex virus type-1 is a double-stranded DNA virus belonging to the *Herpesviridae* family. HSV-1 was the first virus in which TK gene mutation was engineered. In 1991, Martuza et al. demonstrated that human glioblastoma cells can be destroyed by HSV-1 carrying a mutation in the TK region and this was observed in cell culture as well as in nude mice (74). A lot of effort has since been put into making HSV more active against tumor cells and safer for normal cells culminating in the approval of Talimogene laherparepvec (T-Vec), an engineered HSV-1 for the treatment of melanoma in 2015 (75). Phase III trials in patients with non-resectable melanoma showed that T-Vec had higher efficacy in patients with stage IIIB–IV melanoma than GM-CSF treatment alone (76). It was also found that T-Vec in combination with a CTLA-4 inhibitor (Ipilimumab) showed encouraging preliminary results in a phase Ib trial (77), though there is much more work to be done to fully evaluate the effects and outcomes of this treatment combination.

Talimogene laherparepvec has two viral gene deletions (one in the γ34.5 gene and one in the α47 gene) and it has the human GM-CSF gene inserted in place of the deleted γ34.5 gene. The function of γ34.5 is to prevent infected cells from switching off protein synthesis upon viral infection. Considering that tumor cells have a defect in this mechanism, a γ34.5-deleted virus is still able to replicate in these cells. This modified virus is therefore safer as it is only able to replicate in tumor cells. The α47 gene is associated with the downregulation of antigen presentation. A deletion in this region has a double function: first, it is able to increase the anti-tumoral immune response, and second, it is associated with the expression of another gene (US11) which results in boosted viral replication in tumor cells. Finally, the insertion of GM-CSF results in increased anti-tumoral immunity as demonstrated in a phase II study. The study reported an increase in tumor specific CD8^+^ lymphocytes along with a decrease in CD4^+^FoxP3^+^ regulatory T-cells and CD8^+^FoxP3^+^ T-suppressor cells (78, 79).

Another oncolytic HSV is G47Δ, a third generation HSV-1 with three different mutations. It was created by Todo et al. by the deletion of the ICP6 gene from the genome of G207 virus, which already has two mutations (γ34.5 and α47); the *Escherichia coli* LacZ gene was added in place of ICP6 (80). ICP6 encodes the large subunit of ribonucleotide reductase (RR), an enzyme essential for viral DNA synthesis. If this enzyme is missing, the virus fails to replicate. However, tumor cells synthesize a huge amount of RR which can compensate for its deletion from the viral genome. In this way, mutated virus is able to replicate only in tumor cells, becoming safer in normal tissue.

Another example of HSV used as OV is the NV1020 virus which is based on the R7020 construct developed by Meignier et al. (81). NV1020 virus has deletions in the ICP0 and ICP4 gene regions and has only one copy of the γ34.5 gene. Moreover, the α4 promoter which controls TK expression has been inserted, making the virus sensitive to common drugs (such as acyclovir) and improving its safety. This virus has been reported to stabilize metastasis in phase I/II clinical trials involving patients with advanced metastatic colorectal cancer and showed minimal levels of side effects (82).

It has also been found that modification of HSV to include an scFv fragment against HER2 increased viral tropism to HER2 positive tumor cells (83). HSV encodes various glycoproteins to facilitate viral entry and these are: gD, gH/gL, and gB. Usually, receptor recognition leads to modifications in gD and gH/gL which in turn activates gB. Interestingly, this redirected tropism to HER2 positive cells was conferred even when the scFv fragment was engineered into gB (a glycoprotein with a role in virus-cell fusion rather than receptor recognition), thereby bypassing the requirement for receptor mediated activation of gD and gH/gL. This provides a method of improving selectivity and therefore efficacy and safety of oncolytic HSV.

These data indicate a promising route for the clinical use of HSV-1. Many clinical trials have been carried out to treat different types of solid cancer with encouraging results (82, 84, 85). However, some limitations still have to be overcome. A major obstacle for HSV-1 is the way it infects host cells. This virus is able to spread from one cell to another without causing viremia which makes it suitable for intra-tumoral but not intra-venous injection. This could cause some problems in the treatment of tumor lesions which are very difficult to reach directly, like those of pancreatic cancer.

Alongside HSV-1, HSV-2 has also shown promise as an OV. For example, HSV-2 with a deletion of ICP10 to improve selectivity was engineered and found to be even more effective than HSV-1 for treatment of metastatic ovarian cancer in mouse models (86). Building on this initial success, many more studies have been conducted to improve HSV-2 as an OV. These include co-administration of cyclophosphamide, a drug which has chemotherapeutic effects as well as causing a dampening of the innate immune response. This combination works synergistically as reduced innate immunity can facilitate more potent viral infection. Li et al. showed that this combination of therapies leads to enhanced anti-tumor effects when used to treat lung carcinoma in mice and could prove to be a good combination in clinical trials (87). It has also more recently been found that HSV-2 can act in synergy with adoptive T-cell treatment. Administration of oncolytic HSV-2 directly into tumor sites was found to improve the homing of adoptively transferred T-cells (engineered to target tumor cells) to the tumor mass. This was achieved, at least in part, by elevated levels of various chemokines such as CXCL9 and CXCL10 (88, 89). It was also found that in addition to increasing attraction of these T-cells to the tumor, the various chemokines were also able to maintain persistence of these T-cells at the tumor site.

## Newcastle Disease Virus

A more recently developed, naturally oncolytic virus is NDV. It has been found that NDV can effectively kill a variety of tumor cell types and that this activity occurs by induction of immunogenic cell death which in turn leads to adaptive anti-tumor immunity (90).

The initial suggestion of a mechanism for NDV tumor selectivity was that there is a lack of robust/normal anti-viral response in tumor cells. However, it seems that some tumor cells with intact anti-viral pathways are still killed by NDV. It was then found that the lack of apoptosis in tumor cells is what makes NDV tumor-selective (91). Interestingly, it was found that certain strains of NDV can induce apoptosis in tumor cells (92) and that the apoptosis pathway stimulated in infected tumor cells is p53-independent and perhaps triggered by endoplasmic stress (93). It was also found that apoptosis of infected cells occurs predominantly *via* the intrinsic mitochondrial pathway and is caspase dependent (94). This induction of apoptosis results in tumor cell death.

Although NDV is naturally tumor-selective, there are inherent problems with the virus which need to be overcome in order to improve its ability to infect and spread within solid tumors as viral spread is limited by factors such as the extra-cellular matrix (95). Again, there are many studies looking at ways to modify this virus to make it safer and more efficacious; for example, insertion of the IL-2 gene into the NP/P site has proved effective (96). Inclusion of IL-2 and/or TRAIL has been shown to increase apoptosis levels in infected cells resulting in the tumor-selective parental virus becoming an even more potent anti-cancer agent (97–101). Further improvement of oncolytic NDV is found with IL-2 addition in combination with expression of TAA. This combination, delivered by oncolytic NDV, improves tumor-specific T-cell responses leading to higher efficacy than delivery of TAA alone (102). Another strategy to boost the efficacy of oncolytic NDV is to insert the ICOS gene (usually upregulated by viral infection) in order to induce higher levels of T-cell infiltration into the local tumor and distant tumor sites (103).

As well as arming viruses with immune stimulators, other therapeutic genes are also able to increase anti-tumor effects of viral therapies. For example, NDV engineered to encode TNF receptor Fas shows greater oncolytic effect as Fas is responsible for increased apoptosis of infected cells *via* both the intrinsic and extrinsic apoptosis pathways, thereby increasing cell death and in turn anti-tumor efficacy (104). NDV’s naturally oncolytic properties could also be augmented by arming the virus with GM-CSF (105).

In addition to arming the OV with various therapeutic genes and immune stimulators, it is also beneficial to work toward increased virulence (within safety limits). Using a more infectious strain of NDV can produce better cytotoxic effects in tumor cells than arming less virulent strains with immune-modulating genes (106). However, a potential problem encountered with NDV is its ability to infect avian species. This introduces an added difficulty in that any oncolytic therapy involving NDV needs to ensure attenuation of the virus in avian hosts without reduced potency in mammalian cells (107).

It is also important to consider that modification of OVs does not always produce the desired result, for example, viral attenuation to improve safety needs to be carefully tested as production of an attenuated virus which may establish persistent infection could lead to tumors which are not effectively killed by OV and thereby become resistant to treatment. It has previously been reported that NDV has the potential to cause persistent infection in certain cell types (108). This highlights the fact that it is important to carefully engineer OVs in order to maximize efficiency and broaden tropism for a range of cancer types.

Frequently, viral therapies are able to target tumor cells but are not efficacious enough when administered alone or they are potent but toxic at high doses. These problems could potentially be overcome by combining various viral therapies which act synergistically to combat tumor growth. For example, two antigenically distinct viruses which both encode immune stimulators can be sequentially administered and allow two cycles of transgene expression without interference from neutralizing antibodies. This approach allows lower doses to be used (making the treatments safer) and allows a multi-faceted approach which is likely to be more effective than single treatments (69). Oncolytic NDV can also be used in combination with traditional therapies to gain synergistic effects which enhance its action and the overall effect of treatment (109). Also, localized NDV therapy was shown to sensitize distant tumors to treatment with immune checkpoint inhibitors through induction of inflammatory immune infiltrates in these distant sites (110). This study provides a strong basis for developing this combination treatment with potential for entry into clinical trials.

## Retrovirus

In order to overcome the problem of viral infection causing lysis of infected cells and potentially harmful inflammatory responses, a method has been developed whereby retroviral particles which retain their replicative ability can be delivered and will selectively replicate only in cells which are undergoing proliferation (tumor cells) and are compromised in their ability to trigger innate immune responses (again, tumor cells are often unable to trigger innate immunity due to disruption in the signaling pathway). The ability of these particles to integrate into the host genome and replicate without causing lysis of the cell makes them efficient and long lasting producers of the therapeutic gene they are delivering without the consequences of productive viral infection (111).

As is the case for traditional oncolytic viral therapy, this method can be designed using a variety of retroviruses and with the addition of various therapeutic genes. For example, suicide genes which trigger cell death can be delivered to tumor cells *via* particles from various leukemia viruses with varying levels of success (112).

As well as delivering therapeutic genes, replicating retroviral vectors can also be used in order to enhance the response to anti-cancer drug therapy. For example, delivery of an activator of a therapeutic drug by replication competent retroviral vector resulted in significant anti-tumor effect and prolonged survival time in a murine model of malignant mesothelioma (113).

These vectors can also be delivered within a “gutted” Ad genome and the outcome of this combination is improved transfer efficiency of the retroviral genome into the tumor tissue and therefore increased production of the therapeutic gene and better treatment efficacy (114).

## Measles Virus (MV)

Measles virus is a single-stranded, negative sense enveloped RNA virus of the *Paramyxoviridae* family. There are a number of receptors that can be utilized by MV to successfully infect cells including CD150, CD46, and nectin-4. Of these, CD46 has been attributed to increased specificity of MV to tumor cells that express increased levels of this receptor compared with healthy cells. This increased expression leads to increased levels of cell lysis upon infection of tumors compared with healthy tissue (115). Selectivity can also be increased by engineering a MV which is blinded to its usual receptors and redirected to recognize specific tumor cell markers as target antigens (116). Also, as tumor cells often have a defective interferon system they tend to be more susceptible to viral infection leading to increased lysis of these cells by MV in comparison with healthy cells (117). Another method of increasing oncolytic MV selectivity is to engineer miRNA sensitive viruses which can only successfully infect cells in which certain miRNAs are downregulated (i.e., cancer cells). For example, a virus has been developed which shows sensitivity to three host miRNAs through insertion of specific miRNA target sites into the viral genome, rendering the virus incapable of infecting healthy cells which express one or more of these miRNAs but still able to infect specific cancer cells which have downregulated levels of these miRNAs (118).

As well as showing selectivity to tumor cells, oncolytic MV therapy has been shown to recruit certain aspects of the host immune response including neutrophils (119) and dendritic cells (120) to augment tumor cell lysis by also stimulating anti-tumor immune responses to clear the tumor mass. This discovery has prompted development of oncolytic MVs which are engineered to stimulate the immune system at the tumor site in order to exploit the role it plays in anti-tumor immunity. For example, molecules known to stimulate potent immune responses, such as neutrophil-activating protein (NAP) derived from *Helicobacter pylori*, can be engineered into the MV genome to enhance anti-tumor effects generated by oncolytic MV (121). Immune responses can also be manipulated by encoding immune checkpoint blocking antibodies in oncolytic MV genomes. These viruses will result in soluble antibodies against inhibitory immune checkpoints being produced in infected cells which will act to dampen the ability of the immune response to limit itself. Blocking of inhibitory immune checkpoints allows OVs to exploit the immune system to a greater extent than would occur naturally as the immune cells are deprived of negative signals which usually regulate the immune response. This approach has been explored by Engeland et al. with promising results warranting further investigation (122).

One potential problem encountered with oncolytic MV is the widespread immunity in the population, gained from measles vaccinations. This immunity could dampen the effect of oncolytic MV therapy by rapidly clearing the virus before it can take effect on tumor cells. There are a number of potential ways to overcome this, for example, administering immuno-suppressants along with oncolytic MV, however, this approach has drawbacks as immuno-suppression must be carefully managed to ensure patients do not become susceptible to infection by otherwise harmless agents. The most promising solution so far is to “hide” the virus within mesenchymal stem cells, allowing delivery of the virus to the target site without recognition by the immune system and thereby bypassing the effect of neutralizing antibodies (123). It is also possible to exchange measles coat proteins (which are recognized by existing neutralizing antibodies) for those of a virus to which patients are not immune. For example, Miest et al. successfully replaced measles envelope glycoproteins with those from a related virus (canine distemper virus) which allowed the MV genome to be transported without detection by existing neutralizing antibodies against MV envelope proteins (124).

Much progress has been made with MV over the years and this has resulted in many clinical trials being started to ascertain the suitability of oncolytic MV as a clinical treatment. Various cancer types have been targeted for clinical trials of oncolytic MV including myeloma (125) and ovarian cancer (126). Encouraging results have been obtained, especially with regard to safety profiles, and this warrants further studies into optimal oncolytic MV treatments (127).

## Other OVs

Reovirus is a double-stranded, non-enveloped RNA virus of the *Reoviridae* family and is considered a naturally occurring OV. Reoviruses are thought to selectively infect tumor cells because their oncolytic functions depend on the activation of the Ras pathway (128) which tends to be upregulated in transformed cells. Reolysin is a type 3 Reovirus and is so far the only wild-type Reovirus undergoing studies for use as a therapeutic agent. Many clinical trials have been performed or are ongoing and are being conducted on various tumor types as discussed by Gong et al. (129). In 2015, the FDA and EMA granted Reolysin an orphan drug designation for various cancers including gastric, pancreatic, and ovarian cancer (130).

Other viruses are now being tested as therapeutic agents for cancer treatment. As melanoma is easily targeted, many current studies are focused on this neoplasm (131). In recent years, two promising viruses have come out as possible OVs: coxsackievirus and echovirus. The former is responsible for the common cold and enters target cells *via* ICAM-1, while the latter is a positive sense single-stranded RNA virus from the *Picornaviridae* family responsible for many human disorders. As these two strains are the cause of very common diseases in humans, it is very important to ensure their safety.

## Tumor Cell Biology and Viral Therapy

In the search for the most effective viral therapy to treat cancer, alongside the focus on how to improve the virus we can also attempt to influence tumor cell biology to our advantage. This strategy has been adopted by various groups and research has so far shown that there are many tumor cell genes which can be manipulated to increase the efficacy of OVs.

For example, tumor cell genes can play a role in the targeting of OVs to tumor cells. It was found by Cuddington and Mossman that a certain OV (Bovine herpesvirus-1) is better able to infect cells which have increased levels of KRAS expression (e.g., tumor cells) (132). This represents a method of tumor targeting which relies on tumor cell factors to ensure oncolytic therapy is delivered to tumor cells, leaving healthy cells unharmed. This knowledge could potentially be applied to other oncolytic viral therapies by engineering entry mechanisms specific to tumor cells.

The aberrant expression of components of the Raf/MEK/ERK pathway in tumor cells can also have an effect on the regulation of Ad receptor and therefore levels of viral infectivity. As this pathway tends to be upregulated in tumor cells compared with healthy cells, it can have a significant effect on oncolytic viral therapy. It was found that this pathway plays a role in downregulation of CAR (Ad receptor) and therefore oncolytic Ad will be less able to infect target tumor cells. To overcome this, it is possible to inhibit MEK either directly or indirectly in order to inhibit the Raf/MEK/ERK pathway and re-establish expression of CAR on the cell surface (133). However, it has subsequently been shown that phosphorylation of ERK during the later stage of adenoviral infection can actually play a role in facilitating the sustained levels of viral protein within the cell required to produce enhanced levels of progeny virions (134). Taken together, this evidence highlights the need to balance initial inhibition of this pathway to increase CAR expression with later enhancement of the same pathway to facilitate sustained progeny production.

As well as mutations, regulation of certain genes using microRNA can also be used to enhance viral specificity for tumor cells. For example, using an miRNA which is downregulated in tumor cells (such as let-7a) to control expression of an essential viral gene in VV (such as B5R which increases both pathogenicity and oncolytic activity) results in a virus which can only express sufficient amounts of B5R in cells which have low levels of let-7a expression, i.e., tumor cells (15).

Another gene found in tumor cells that can influence OV therapy is VEGF. Our group has demonstrated that VEGF-A increases VV internalization and in turn replication levels (135). Therefore, oncolytic VV can take advantage of the increased expression of VEGF by tumor cells to increase delivery of therapeutic genes which in turn increases the efficacy and potency of the treatment. In addition to this, Arulanandam et al. found that the increase in VEGF expression upon infection with VV leads to upregulation of PRD1-BF1 (a transcription repressor) which increases sensitivity of tumor vascular endothelial cells to infection with vaccinia *via* repression of type-1 interferon anti-viral signaling. This increase in viral tropism in turn allows the OV to spread through the tumor more efficiently and therefore increases the efficacy of this oncolytic therapy (136). This natural repression of interferon signaling highlights the potential of using interferon inhibitors to increase the efficacy of oncolytic viral therapy, as seen by Stewart et al. (137).

It has also been found that a properly functioning host interferon response pathway is a critical factor in measles infection of malignant pleural mesothelioma. It was seen that in cell lines, there is a correlation between sensitivity of cells to measles infection and an inability of the cell to elicit a full interferon response in the presence of MV (138). This warrants further investigation as it suggests that inhibition of the interferon pathway could prove to be critical in ensuring the efficiency of oncolytic therapy. Previous to this study, it was also found that VV infection is greatly increased through downregulation of c-Jun NH2-terminal kinase (JNK). Inhibition of the JNK signaling cascade leads to lower levels of double-stranded RNA dependent protein kinase which in turn allows increased replication of VV genomes (139). This knowledge provides an avenue of exploration for improvement of OV efficacy.

Also, it has been reported that another gene found in tumor cells (CEACAM6) has an effect on oncolytic viral therapy. This tumor-associated gene has various functions including a role in promotion of tumor adhesion and invasion among other factors (140). It was shown by Wang et al. that over-expression of CEACAM6 did not have an effect on Ad receptor expression or at attachment and internalization steps of infection but did interfere with cytoplasmic virus trafficking to the nucleus *via* reduced expression of cytoskeletal proteins (141). As this cell adhesion molecule is able to inhibit adenoviral infection of tumor cells, systemic pre-treatment with siRNA targeting this protein could significantly enhance the anti-tumor response generated by adenoviral vectors.

Another host system which can have a significant effect on oncolytic viral efficacy is the stress response pathway. In the case of oncolytic rhabdovirus, inhibition of certain ER stress response factors can significantly increase efficacy of subsequently delivered oncolytic rhabdovirus (142). This method of pre-conditioning tumor cells to improve subsequent viral infection warrants further study as a potential method of increasing efficacy of oncolytic therapy in patients.

In addition to this, induction of the unfolded protein response in tumor cells has been found to increase the efficacy of oncolytic Ad by improving viral spread and tumor cell killing (143).

Recently, it has been reported that host microRNAs are able to regulate infection of cells with various viruses and in various ways. One of these is the control of Ad replication by miR-27a/b which downregulates SNAP25 and TXN2. This leads to a reduction in Ad entry into cells and cell cycle arrest, respectively (which in turn reduce replication of Ad within the cell) (144). Controlling expression of host miRNA by inhibition of miRNA processing factors could therefore be a promising way to ensure maximal efficacy of oncolytic Ad therapies.

These examples open new insight in the field of oncolytic viral therapy revealing the possibility to manipulate virus–host interaction. We are still far from the optimum and new studies should be focused on the long-term effects of this interaction and the possible side effects.

## Conclusion and the Future of the Field

Oncolytic viral therapy is a promising treatment for cancer. New knowledge regarding both viral biology and tumor cell biology has made it possible to improve several aspects of OV therapy including safety, potency, and delivery methods. Also, the possibility of relying not only on direct lysis of tumor cells by viral infection but also mounting a multi-faceted approach involving viral lysis, immune stimulation, and gene therapy has become an important aspect of research in this field.

As the current treatment options for cancer tend to rely on only one method of attack, formulating a therapy which has the ability to act in multiple ways against cancer is an extremely exciting prospect. In order to effectively kill tumor cells and also reduce the chance of recurrence, it is necessary to utilize both the ability of virus infection to lyse the cells which are infected along with the ability of virus infection to stimulate a potent immune response, generating long lasting immunity against the antigens present on tumor cells.

If we can also bring into play gene therapy which can act in multiple ways, ultimately leading to reduction in tumor volume, we can make oncolytic viral therapy an even more formidable weapon in the fight against cancer.

The next step in this area should be to improve efficacy through arming with immuno-modulatory genes. These genes can influence the host response to viral infection, stimulating long-term immunity in the form of memory T-cells and attracting immune cells to the tumor mass through stimulation of cytokine production. In addition, combined therapy with conventional agents should be another relevant field of research, in order to increase viral action. This area of research involves a multitude of possibilities including ways to increase the host response to virus (for example, blocking immune checkpoints) and also the use of oncolytic therapy to augment the action of conventional therapy like chemotherapy and radiotherapy.

We also need to focus on exploiting the range of genes found to be differentially expressed in tumor cells which have recently been seen to play a role in potency of OVs. If we are able to gain a better understanding of the interaction between virus and tumor cells, we can overcome some of the obstacles in OVT by modulating the expression of tumor cell genes to enhance viral infectivity and efficacy.

Ultimately, the long-term goal is to formulate a treatment which can target solid tumors and circulating tumor cells in a number of ways simultaneously (145), with the aim of mounting a response that is effective even if the tumor cells become resistant to one approach. In order to do this, we need to take into consideration the interactions between tumor cells, virus, and the host. The most effective treatments will be designed to improve viral infection of tumor cells, boost immune responses to tumor antigens and manipulate tumor cell gene expression and pathways to favor successful viral infection. This can improve on current therapies which are ineffective once the tumor cells gain resistance, an issue which occurs often given the heterogeneous and rapidly mutating nature of many cancers.

## Author Contributions

YW conceived the study, interpreted the data, and finalized the manuscript. AH and GM designed the study, searched references, collected data, and wrote the manuscript. NL participated in interpretation of some data and critically reviewed the manuscript.

## Conflict of Interest Statement

The authors declare that the research was conducted in the absence of any commercial or financial relationships that could be construed as a potential conflict of interest.
